# Metacognitive Beliefs Uniquely Contribute to Interpersonal Problems: A Test Controlling for Adult Attachment, Big-5 Personality Traits, Anxiety, and Depression

**DOI:** 10.3389/fpsyg.2021.694565

**Published:** 2021-09-01

**Authors:** Henrik Nordahl, Odin Hjemdal, Adrian Wells

**Affiliations:** ^1^Department of Psychology, Norwegian University of Science and Technology, Trondheim, Norway; ^2^School of Psychological Sciences, Faculty of Biology, Medicine and Health, The University of Manchester, Manchester, United Kingdom; ^3^Greater Manchester Mental Health NHS Foundation Trust, Manchester, United Kingdom

**Keywords:** interpersonal problems, personality, traits, attachment, metacognitive beliefs, metacognition, S-REF model, metacognitive control system

## Abstract

Interpersonal difficulties are common across psychological disorders and are a legitimate target of treatment. Psychotherapeutic models differ in their understanding of interpersonal problems and how these problems are formulated and treated. It has been suggested that they are both the cause and effect of emotional distress symptoms, that they result from early attachment experiences, and that they are related to personality dimensions. However, the metacognitive model of psychopathology predicts that emotion disorder symptoms and interpersonal problems are linked to a common set of factors involving dysfunctional metacognition. In support of this view, metacognitive therapy has substantially reduced interpersonal problems in patients with anxiety and depression even though interpersonal problems are not directly targeted, indicating a role for metacognitive change. Nevertheless, the relationship between interpersonal problems and metacognitive beliefs remains underexplored, and the statistical control of emotion symptoms, personality, and attachment is important in substantiating any metacognition effects. The aim of the present study was therefore to test metacognitive beliefs as statistical predictors of interpersonal problems while controlling for anxiety/depression, adult attachment, and the Big-5 personality dimensions. In a cross-sectional study, 296 participants completed a battery of self-report questionnaires. We found that positive- and negative-metacognitive beliefs, cognitive confidence, and cognitive self-consciousness accounted for significant and unique variance in interpersonal problems together with avoidant attachment and conscientiousness when the overlap between all predictors was controlled. These findings support the notion that metacognitive beliefs are relevant to interpersonal problems with the potential implication that metacognitive therapy could have particularly broad effects on both emotion disorder symptoms and interpersonal problems.

## Introduction

Interpersonal problems are central to personality disorders (Hopwood et al., 2013; Wilson et al., 2017) and are common in axis-I disorders including anxiety disorders (Eng and Heimberg, 2006; Cain et al., 2010; Tonge et al., 2020), post-traumatic stress (Elmi and Clapp, 2021), obsessive compulsive disorder (Solem et al., 2015), eating disorders (Hartmann et al., 2010; Arcelus et al., 2013), and major depressive disorder (Bird et al., 2018). They are frequent complaints in those seeking psychotherapy (Horowitz et al., 1988) and predict less improvement in therapy and greater dropout (Hilbert et al., 2007; Renner et al., 2012; Dinger et al., 2013; Quilty et al., 2013; McEvoy et al., 2014; Newman et al., 2017). Specific domains of interpersonal problems appear to be more strongly associated with some disorders than others (i.e., interpersonal prototypicality), but there is also evidence for heterogeneity within groups (Girard et al., 2017; Shin and Newman, 2019). Nonetheless, improved overall interpersonal functioning is an important goal for psychotherapeutic interventions, and this objective brings a greater need to understand how interpersonal problems are initiated and maintained.

Several factors may contribute to interpersonal problems. For example, interpersonal problems show a reciprocal relationship with emotional distress symptoms (Grant et al., 2013; Hepp et al., 2017), indicating that they may be both the cause and effect of reduced ability to deal with negative affect and stressors. From a developmental perspective, attachment experiences have been suggested as etiological factors underlying interpersonal functioning in general (Bowlby, 1973). In their systematic review, Hayden et al. (2017) reported that an adult attachment style of attachment anxiety and avoidance is associated with more interpersonal problems. There is also evidence for an association between the Big-5 personality traits (Costa and McCrae, 1985) and interpersonal problems. For example, interpersonal problems are positively and significantly correlated with neuroticism and negatively correlated with extraversion and openness (Nysæter et al., 2009). More nuanced analyses of the relationships between the Big-5 and interpersonal problems indicate that the personality dimensions are consistently associated with interpersonal functioning even at the facet level, and it has been argued that integrating personality profiles with profiles of interpersonal functioning can facilitate personality assessment (Du et al., 2020).

It is important that research takes into account overlap in constructs, where anxious and avoidant attachment, elevated neuroticism and interpersonal problems may simply reflect the presence of emotional disorder symptoms. Furthermore, these factors may be related to a common underlying mechanism. In particular, the metacognitive model of psychological disorders (Wells and Matthews, 1994; Wells, 2019) is based on the principle that emotion disorder and interpersonal problems are caused by a pattern of processing and self-regulation termed as the cognitive attentional syndrome (CAS), which consist of perseverative negative thinking such as worry and rumination, threat monitoring, and unhelpful coping behaviors. Individual differences in CAS activity are linked to biases in the persons metacognitive control system, marked by particular metacognitive beliefs. Metacognitive beliefs are seen as a transdiagnostic factor underlying different types of emotional distress and psychopathologies, with negative metacognitive beliefs about the uncontrollability and danger of cognition viewed as especially important (Wells, 2009). Negative metacognitive beliefs compromise self-regulation by prohibiting disengagement from the CAS and contributing to negative and threatening interpretations of cognitive events. Thus, in the presence of dysfunctional metacognitions, a person may struggle to regulate their thinking in an adaptive way and rely on interpersonal strategies as a means to achieve cognitive regulation. For example, believing that worrying is uncontrollable and dangerous can prohibit internal disengagement from worrying and instead the individual seeks control through excessive reassurance seeking, avoidance of social situations, self-injurious behaviors, or aggressive responses that aim to manage the environment but have a particular interpersonal signature. To take another example, a negative belief about cognitive efficiency might lead to in-situation social behaviors aimed to compensate such as acting with superiority and disregard for other’s opinions or conversely not engaging with tasks at all.

Whilst there is now robust evidence that metacognitive beliefs significantly correlate with emotional distress symptoms across disorders (Sun et al., 2017), with psychological vulnerability (Nordahl et al., 2019), and with the Big-5 personality traits (Marino et al., 2016), the association between metacognitive beliefs and interpersonal problems is under-explored. There is some evidence that metacognitions are associated with interpersonal problems in the form of social avoidance even when controlling social phobic cognitive beliefs (e.g., Nordahl et al., 2017) and metacognitive therapy (MCT; Wells, 2009), specifically developed to modify metacognitive knowledge is an effective treatment for patients with anxiety and depression (Normann and Morina, 2018), and it is associated with improvements in interpersonal problems in patients with generalized anxiety (Nordahl et al., 2018), major depressive disorder (Hjemdal et al., 2017; Strand et al., 2018), social anxiety (Nordahl et al., 2016), and borderline personality (Nordahl and Wells, 2019). These indirect results are consistent with the hypothesis that metacognitive beliefs are associated with interpersonal problems, but such relationships remain to be directly tested under rigorous conditions that control for emotion symptoms and personality and adult attachment styles. This was our aim in the present study.

In line with previous research, we hypothesized that higher levels of anxiety and depression, higher endorsement of insecure adult attachment styles, higher neuroticism, and lower extraversion and agreeableness would be significantly associated with greater interpersonal problems. We also hypothesized that metacognitions would be positively and significantly correlated with interpersonal problems and that metacognitive beliefs would explain unique variance in interpersonal problems when controlling for all other pre-specified predictors. This is because the metacognitive model identifies a common underlying set of metacognitions involved across psychological disorders that are marker of bias in a separate metacognitive system. Among the metacognition domains, we further expected that negative metacognitive beliefs about the uncontrollability and danger of worry would be a specific statistical predictor of interpersonal problems, because this belief domain is central in mental regulation and individuals endorsing such beliefs are likely to use other strategies (e.g., interpersonal strategies) to self-regulate leading to interpersonal problems.

## Materials and Methods

### Participants and Procedure

Two-hundred and ninety-six (*N*=296) participants took part in the study and were gathered at convenience at a lecture for undergraduate psychology students. All participants were Caucasian and Norwegian was their native language. Two-hundred and thirty (77.7%) of the included participants were female. The mean age of the overall sample was 22.35 (SD=3.29) years old. All participants signed a written informed consent prior to taking part in the study, and the study was approved by the Regional Committee for Medical and Health Research Ethics in Norway (REC: 2012/55).

### Measures

#### Interpersonal Problems

The inventory of interpersonal problems (Horowitz et al., 1988) was developed as a measure of difficulties people experience in their interpersonal relationships and originally consisted of 127 items. It assesses things people think they do too much (e.g., get irritated) and things they find hard to do (e.g., participate in groups). Each item is rated on a five-point scale ranging from 0 (“not at all”) to 4 (“extremely”) and can be scored on eight octants (scales) that are arranged around a circumplex (Alden et al., 1990). These scales are as follows (Alden et al., 1990): (1) Domineering, reflecting interpersonal problems caused by overly dominant behavior (e.g., “I try to control others too much”); (2) Vindictive, reflecting interpersonal problems related to distrust and suspicion of others and an inability to care about others’ needs and happiness (e.g., “It is hard for me to trust other people”); (3) Cold, reflecting problems with expressing affection toward and to feel love for another person, difficulty making long-term commitments to others, and an inability to be generous to, get along with, and forgive others (e.g., “It is hard for me to get along with people”); (4) Socially Avoidant, reflecting problems with social interactions, expressing feelings, and socializing with others (e.g., “It is hard for me to ask other people to get together socially with me”); (5) nonassertive, reflecting difficulty making needs known to others, discomfort in authoritative roles, and inability to be firm with and assertive toward others (e.g., “It is hard for me to be assertive with another person”). (6) Exploitable, reflecting problems with feeling anger and expressing anger for fear of offending others and the tendency to be taken advantage of by others (e.g., “I am too easily persuaded by other people”); (7) Overly Nurturant, reflecting the extent one tries to please others and is too caring and permissive in dealing with others (e.g., “I put other people’s needs before my own too much”); and (8) Intrusive, reflecting inappropriately self-disclosing, attention seeking, and problems with spending time alone (e.g., “I want to be noticed too much”). Previous research on IIP has shown a general agreement that the instrument taps several types of interpersonal problems, but it has also shown disagreement as to exactly how many distinct dimensions are represented. Nonetheless, a global distress score (sum/mean of all items) of interpersonal problems can also be used as a non-specific indicator of interpersonal distress. There are several shortened versions of the IIP, and in the current study, we used the 48-item version which has been validated in a Norwegian sample (Gude et al., 2000). In the current study, we focused on the global distress score of which the alpha was 0.91.

#### Anxiety and Depression

The Hopkins Symptom Checklist-25 (Derogatis et al., 1974) is a 25-item self-report scale measuring anxiety and depression symptoms over the past 2weeks. Responses for each item are required on a four-point scale ranging from 1 (“not at all”) to 4 (“extremely”). Higher scores reflect higher levels of emotional distress, and the scale can be subdivided into anxiety symptoms (first 10 items) and depression symptoms (last 15 items). The HSCL-25 has been validated in Norwegian and shown good internal consistency with an alpha of 0.93 for the total scale (Strand et al., 2003). In the current study, the Cronbach alpha for the anxiety subscale was 0.79 and 0.90 for the depression subscale.

#### Attachment

The measure of attachment qualities (Carver, 1997) is a self-report measure of adult attachment based on the four-category model of attachment (Bowlby, 1973; Bartholomew and Horowitz, 1991); Secure (e.g., “It feels relaxing and good to be close to someone”), Avoidant (e.g., “I prefer not to be too close to others”), Ambivalence-worry (e.g., “I often worry that my partner does not really love me”), and Ambivalence-merger (e.g., “I find that others are reluctant to get as close as I would like”). Items are scored on a four-point scale ranging from 1 (“disagree a lot”) to 4 (“agree a lot”). The validity of the original MAQ was established by correlating it with two other attachment measures such as the Relationship Questionnaire (Bartholomew and Horowitz, 1991). The internal consistency for the MAQ has been reported as adequate with Cronbach alphas for the subscales ranging from 0.69 to 0.76 (Carver, 1997). In the current study, the Cronbach alphas for the four factors were 0.70 for secure, 0.78 for avoidant, 0.82 for ambivalence-worry, and 0.27 for ambivalence-merger.

#### Personality Traits

The NEO-PI-R (Costa and McCrae, 1992) contains 240 items, grouped into 30 facet scales that are hierarchically organized under five domain scales corresponding to the five-factor model and thus assess the following traits; Neuroticism (N), Extraversion (E), Openness (O), Agreeableness (A), and Conscientiousness (C). Responses are required on a five-point scale ranging from 0 (“strongly disagree”) to 4 (“strongly agree”). The NEO-PI-R has been validated in Norwegian and shown good internal consistency with alphas ranging from 0.85 to 0.93 in a community sample (Martinsen et al., 2005). In the current study, the Cronbach alphas for the five factors were N=0.83, E=0.81, O=0.65, A=0.65, and C=0.82.

#### Metacognitions

The metacognitions questionnaire 30 (MCQ-30; Wells and Cartwright-Hatton, 2004) is a 30-item self-report scale measuring metacognitive beliefs (i.e., beliefs about cognition) related to the metacognitive model. Responses are required on a four-point scale ranging from 1 (“do not agree”) to 4 (“agree very much”). Higher scores reflect stronger endorsements of the beliefs in question. The MCQ-30 has a five-factor structure concerning: (1) positive beliefs about worry (POS), (2) negative beliefs about the uncontrollability and danger of worry (NEG), (3) cognitive confidence (CC), (4) need to control thoughts (NC), and (5) cognitive self-consciousness (CSC). The measure has been validated in Norwegian and shown good internal consistency with *α* ranging from 0.79 to 0.88 in a community sample (Nordahl et al., 2019). In the current study, the Cronbach alphas for the five subscales were POS=0.78, NEG=0.83, CC=0.80, NC=0.65, and CSC=0.82.

### Overview of Statistical Analyses

Pearson bivariate correlations were used to explore the relationships between interpersonal problems, anxiety, depression, the four types of attachment, the Big-5 personality traits, and the five domains of metacognitive beliefs. A hierarchical linear regression was run to test the relative importance of gender/age, anxiety/depression, attachment styles, personality traits, and metacognitive belief domains to interpersonal problems. We pre-specified the predictor variables as follows: gender and age were entered in the first step, anxiety and depression in the second step, the four attachment styles in the third step, the five personality dimensions in the fourth step, and the five metacognitive belief domains were entered in the fifth and final step.

## Results

### Correlational Analyses

Interpersonal problems were significantly and positively correlated with anxiety, depression, the four attachment styles, neuroticism, and all the metacognitive belief domains as we had predicted. Furthermore, interpersonal problems were significantly and negatively correlated with Extraversion and Conscientiousness, but there was no significant correlation with Openness or Agreeableness. The bivariate correlations and the means and standard deviations for all variables are presented in Table 1.

**Table 1:** Bivariate correlations, means and standard deviations (*N*=296).

		2	3	4	5	6	7	8	9	10	11	12	13	14	15	16	17	M (SD)
1.	IP	0.465^**^	0.469^**^	0.237^**^	0.384^**^	0.388^**^	0.379^**^	0.500^**^	−0.265^**^	−0.031	−0.042	−0.269^**^	0.282^**^	0.515^**^	0.391^**^	0.285^**^	0.259^**^	40.79 (19.55)
2.	Anxiety		0.759^**^	0.023	0.112	0.438^**^	0.320^**^	0.624^**^	−0.138^*^	0.057	−0.019	−0.147^*^	0.149^*^	0.633^**^	0.332^**^	0.297^**^	0.247^**^	14.49 (3.79)
3.	Depression			0.143^*^	0.266^**^	0.422^**^	0.348^**^	0.654^**^	−0.293^**^	−0.075	−0.065	−0.194^**^	0.135^*^	0.617^**^	0.352^**^	0.342^**^	0.177^**^	22.51 (7.22)
4.	MAQsec				0.568^**^	−0.002	0.157^**^	0.083	−0.397^**^	−0.243^**^	−0.194^**^	−0.114^*^	0.110	0.003	0.047	−0.007	−0.004	4.71 (1.49)
5.	MAQavo					0.242^**^	0.290^**^	0.223^**^	−0.465^**^	−0.184^**^	−0.107	−0.129^*^	0.135^*^	0.174^**^	0.197^**^	0.072	0.095	11.18 (2.77)
6.	MAQa-wo						0.475^**^	0.529^**^	−0.219^**^	−0.056	−0.034	−0.206^**^	0.166^**^	0.478^**^	0.245^**^	0.297^**^	0.093	6.42 (2.11)
7.	MAQa-me							0.329^**^	−0.147^*^	0.008	−0.076	−0.141^*^	0.245^**^	0.314^**^	0.294^**^	0.179^**^	0.129^*^	6.07 (1.43)
8.	N								−0.288^**^	−0.046	−0.139^*^	−0.295^**^	0.178^**^	0.634^**^	0.327^**^	0.285^**^	0.100	53.01 (9.74)
9.	E									0.393^**^	0.147^*^	0.172^**^	−0.112	−0.190^**^	−0.157^**^	−0.066	−0.069	51.64 (10.59)
10.	O										0.139^*^	0.038	−0.053	0.083	−0.046	−0.067	0.197^**^	54.01 (9.12)
11.	A											0.027	−0.104	−0.023	−0.024	0.041	−0.095	50.56 (9.94)
12.	C												0.030	−0.031	−0.329^**^	−0.037	0.220^**^	48.04 (10.77)
13.	MCQpos													−246^**^	0.078	0.303^**^	0.296^**^	8.73 (2.63)
14.	MCQneg														0.275^**^	0.423^**^	0.393^**^	10.27 (3.80)
15.	MCQcc															0.229^**^	0.098	9.95 (3.47)
16.	MCQnc																0.393^**^	8.95 (2.67)
17.	MCQcsc																	12.70 (3.93)

M, mean; SD, standard deviation; IP, interpersonal problems; MAQsec, measure of attachment qualities – security; MAQavo, measure of attachment qualities – avoidance; MAQa-wo, measure of attachment qualities – ambivalence-Worry; MAQa-me, measure of attachment qualities – ambivalence-merger; N, neuroticism; E, extraversion; O, openness; A, agreeableness; C, conscientiousness; MCQpos, positive metacognitive beliefs; MCQneg, negative metacognitive beliefs; MCQcc, cognitive confidence; MCQnc, need for control; MCQcsc, cognitive self-consciousness.

* *p*<0.05;

** *p*<0.01.

### Linear Regression Analyses

Interpersonal problems indicated by the total score of the IIP-48 were treated as the dependent variable. In the first step, gender and age entered as a block were non-significant as predictors, and neither of them accounted for unique variance in interpersonal problems. In the second step, anxiety and depression were entered as a block and accounted for an additional 23.6% of the variance and each contributed independently. In the third step, the block of attachment styles was entered, resulting in an additional 11.7% of the variance explained. In this step, ambivalence-worry and ambivalence-merger made individual contributions, while security and avoidance did not. In the fourth step, the Big-5 personality dimensions were entered and together accounted for an additional 4.2% of the variance. Among the personality dimensions, neuroticism and conscientiousness made individual contributions. In the fifth and final step, metacognitive belief domains were entered as a block and made a significant additional contribution, accounting for 7.9% of the variance. In the final model, when controlling for the overlap between all the predictors, higher scores on the avoidant attachment style, lower conscientiousness, higher levels of beliefs about uncontrollability and danger of worry, and lower confidence in memory (i.e., higher cognitive confidence score denotes poorer confidence) significantly and independently contributed. Statistics for regression model are summarized in Table 2.

**Table 2:** Hierarchical linear regression with interpersonal problems as the dependent, and gender, age, attachment, personality, and metacognitions as predictors (*N*=296).

Step	Variables	*F* change	*R*^2^ change	*β*	*t*
1		1.915	0.013		
	Gender			−0.08	−1.352
	Age			−0.09	−1.527
2		45.768	0.236^**^		
	Gender			0.01	0.167
	Age			−0.07	−1.378
	Anxiety			0.24	3.071^**^
	Depression			0.28	3.621^**^
3		13.194	0.117^**^		
	Gender			−0.01	−0.217
	Age			−0.05	−0.967
	Anxiety			0.25	3.234^**^
	Depression			0.12	1.557
	MAQsec			0.07	1.122
	MAQavo			0.22	2.496^**^
	MAQa-wo			0.12	1.997^*^
	MAQa-me				
4		4.044	0.042^**^		
	Gender			0.03	0.593
	Age			−0.07	−1.515
	Anxiety			0.20	2.590^*^
	Depression			0.03	0.347
	MAQsec			0.06	1.004
	MAQavo			0.21	3.338^**^
	MAQa-wo			0.05	0.771
	MAQa-me			0.13	2.368^*^
	N			0.23	3.094^**^
	E			0.01	0.143
	O			0.02	0.296
	A			0.05	1.009
	C			−0.12	−2.384^*^
5		8.560	0.079^**^		
	Gender			−0.03	−0.595
	Age			−0.09	−1.836
	Anxiety			0.09	1.152
	Depression			−0.01	−0.114
	MAQsec			0.09	1.546
	MAQavo			0.16	2.753^**^
	MAQa-wo			0.02	0.412
	MAQa-me			0.08	1.521
	N			0.12	1.628
	E			0.03	0.502
	O			−0.03	−0.551
	A			0.06	1.240
	C			−0.17	−3.342^**^
	MCQpos			0.13	2.612^**^
	MCQneg			0.20	2.919^**^
	MCQcc			0.14	2.788^**^
	MCQnc			−0.01	−0.104
	MCQcsc			0.12	2.062^*^

MAQsec, measure of attachment qualities – security; MAQavo, measure of attachment qualities – avoidance; MAQa-wo, measure of attachment qualities – ambivalence-Worry; MAQa-me, measure of attachment qualities – ambivalence-merger; N, neuroticism; E, extraversion; O, openness; A, agreeableness; C, conscientiousness; MCQpos, positive metacognitive beliefs; MCQneg, negative metacognitive beliefs; MCQcc, cognitive confidence; MCQnc, need for control; MCQcsc, cognitive self-consciousness.

* *p*<0.05;

** *p*<0.01.

## Discussion

To the authors’ knowledge, this is the first study to investigate metacognitive beliefs as statistical predictors of interpersonal problems and to evaluate their relative contribution when controlling for gender/age, anxiety, depression, adult attachment, and the Big-5 personality dimensions. Investigating the contributions of these variables is important for identifying psychological factors that might be assessed in future modeling of interpersonal problems. Such factors might be indicative of potential targets for treatment (e.g., anxiety, depression, and metacognitions), but their effects must be separated from potential influences of personality dimensions and attachment styles with which they also correlate.

We observed significant bivariate correlations between most of the measures, and as predicted there were significant associations between all MCQ-30 subscales and severity of interpersonal problems. Here the strongest association was with uncontrollability and danger beliefs (*r*=0.515) and the weakest was with CSC (*r*=0.259). These results are consistent with the prediction that metacognitions are related to interpersonal problems and that negative beliefs about control and danger of worry may be particularly important in this relationship.

The results of the regression analysis add further evidence supporting a relationship between metacognitive beliefs and interpersonal problems by controlling for a range of variables. In the final model, one out of five personality dimensions individually contributed, compared with four out of five of the metacognition variables. When controlling for the overlap between all the variables in the final step, six out of the 18 predictors made a unique and significant statistical contribution overall to interpersonal problems: higher scores on avoidant attachment style, lower conscientiousness score, and from the metacognition subscales higher scores in positive beliefs, negative beliefs, cognitive confidence and CSC. Thus, gender/age, anxiety/depression, secure and ambivalent attachment styles, neuroticism/extraversion/openness/agreeableness, and need for control were non-significant as predictors of interpersonal problems in this model. Among the significant predictors, negative metacognitive beliefs were the strongest unique predictor.

This is a potentially important set of findings because it suggests that metacognitive beliefs might make a substantive individual contribution to interpersonal problems (at least cross-sectional, i.e., at a maintenance level) and the effect is not dependent on the other variables measured. Of particular note, an incidental finding is that the majority of the Big-5 personality dimensions did not contribute independently to interpersonal problems, with only conscientiousness making a significant and negative contribution. This is a prominent finding in the context of the current literature on personality and interpersonal functioning as it implies that metacognition rather than individual differences in personality might be more consistent direct correlates of interpersonal problems.

The finding of a contribution of a range of metacognition subscales with an emphasis on negative beliefs about uncontrollability in particular fits with the metacognitive model of psychological disorder (Wells and Matthews, 1994; Wells, 2019) and potentially extends the model to understanding mechanisms of interpersonal dysfunction. In the model, beliefs concerning the uncontrollability and dangerousness of worrying are viewed as part of a common mechanism involving biased and unhelpful patterns of cognitive regulation. For example, cognitive regulation can be achieved through choice of interpersonal strategies such as seeking reassurance to reduce worry or avoidance of interpersonal closeness (e.g., hostility/avoidance) to reduce the triggering of thoughts about rejection. Of note, the present analysis also showed contributions of elevated positive beliefs about worrying, lower levels of confidence in cognition (i.e., memory), and higher CSC. Overall, this pattern would be consistent with a range of different metacognitive biases contributing to interpersonal problems, with negative beliefs about control and the dangerousness of thoughts demonstrating the strongest independent effect. There are several implications of this finding: (1) the metacognitive model may be applicable to formulating interpersonal problems, (2) it may be necessary to modify dysfunctional metacognitions to improve interpersonal problems, (3) interpersonal problems may be responsive to MCT, and (4) future research on interpersonal problems should take account of the effects of metacognition.

Attachment in the form of an avoidant style was a significant and unique statistical predictor of interpersonal problems in the final regression equation. This is in line with previous research (Hayden et al., 2017) but in interpreting the finding it should be noted that the items assessing this type of attachment style are very similar to the IIP-items (e.g., “I prefer not to be too close to others”; “I get uncomfortable when someone wants to be very close”), so the significant contribution from avoidant attachment might be explained by content overlap in the measures rather than being theoretically substantive. Secure attachment unexpectedly correlated positively with interpersonal problems but did not account for unique variance when the other attachment styles were controlled.

Only one personality dimension, conscientiousness uniquely correlated with interpersonal problems. Here lower conscientiousness was associated with an increase in interpersonal problems. This is in line with previous research indicating that higher levels of conscientiousness are associated with lower interpersonal problems overall and that conscientious individuals tend to be good at being warm, trusting others, and being gregarious (Du et al., 2020). However, our findings that the other personality dimensions did not account for unique variance in interpersonal problems are not in line with previous research, which has reported that neuroticism, extraversion, and agreeableness are the strongest predictors of interpersonal problems (e.g., Nysæter et al., 2009). But in light or our findings, it is notable that these other studies did not control for metacognition and attachment variables. It could be that metacognitive beliefs partially account for the association between several personality dimensions and interpersonal problems. For example, we observed that neuroticism was significantly related to interpersonal problems in step four, but that this relationship was non-significant in step five when metacognitive beliefs entered, which may indicate that neuroticism is a surface marker for maladaptive metacognitive beliefs as reported in previous studies (Hjemdal et al., in prep; Nordahl et al., 2019).

Recent studies have shown that MCT (Wells, 2009), which targets metacognitive beliefs and mental regulation, is associated with positive effects on interpersonal problems in patients with primary anxiety and depression disorders (Nordahl et al., 2016, 2018; Strand et al., 2018) and with borderline personality disorder (Nordahl and Wells, 2019). Thus, modifying metacognitions in treatment may create change in interpersonal problems, even without directly aiming to target them. MCT could provide a common pathway for dealing with both emotion disorder symptoms and interpersonal difficulties, providing a unitary framework for improving multiple dimensions of self-regulation and social functioning.

Incidental to the primary aims of the current study, we observed some interesting correlations between metacognitions and attachment that are worth mentioning. While dysfunctional metacognitive belief domains did not significantly correlate with secure attachment, there were correlations with insecure attachment. Positive metacognitive beliefs, negative metacognitive beliefs, and cognitive confidence were positively and significantly associated with all three types of insecure attachment. Need for control positively and significantly correlated with ambivalence-worry and ambivalence-merger, and CSC positively and significantly correlated with ambivalence-merger. These observations are in line with a previous study that reported a relationship between metacognitive beliefs and anxious attachment style (Myers and Wells, 2015) and suggests that further research should investigate the relationships between the metacognitive model (Wells, 2019) and attachment theory. The results support the call for future studies that examine the development of metacognitions as specified in the metacognitive model (Wells, 2019), with insecure attachments offering a candidate risk factor for dysfunction in the metacognitive control system.

This study has several limitations that should be acknowledged and considered in any interpretation. First, the sample was gathered at convenience and consisted of substantially more females than males. Second, due to the cross-sectional design of the study, causal inferences cannot be tested. Third, all data relied on self-report measures which may not give an unbiased assessment. Forth, some of the scales included in our analyses showed low internal consistency. This was particularly so for the ambivalence-merger subscale of the MAQ and we do not know why this was the case. Hence, we must be cautious in interpreting and generalizing from these findings. Nonetheless, our study provides initial evidence indicating a role for metacognitions in interpersonal problems while controlling for other important factors. Further research should investigate these relationships in clinical samples, using alternative assessment tools and specifically examining the role of metacognitions in different domains of interpersonal problems. Domains of metacognitive beliefs not explored in the current study may also contribute to interpersonal problems, such as negative metacognitive beliefs about the interpersonal and social consequences of rumination (Papageorgiou and Wells, 2001). In addition, further research should investigate the associations between metacognitions and interpersonal problems in longitudinal data to test for temporal relations. In conclusion, we observed specific associations between metacognitions and interpersonal problems that are consistent with the metacognitive model of psychological disorders and were independent of personality, emotion disorder symptoms and attachment styles.

## Data Availability Statement

The raw data supporting the conclusions of this article will be made available by the authors, without undue reservation.

## Ethics Statement

The studies involving human participants were reviewed and approved by the Regional Committees for Medical and Health Research Ethics. The patients/participants provided their written informed consent to participate in this study.

## Author Contributions

HN, OH, and AW planned the study and wrote the manuscript. OH collected the data. AW and HN planned the statistical analysis. OH and HN analyzed the data. All authors contributed to the article and approved the submitted version.

## Conflict of Interest

The authors declare that the research was conducted in the absence of any commercial or financial relationships that could be construed as a potential conflict of interest.

## Publisher’s Note

All claims expressed in this article are solely those of the authors and do not necessarily represent those of their affiliated organizations, or those of the publisher, the editors and the reviewers. Any product that may be evaluated in this article, or claim that may be made by its manufacturer, is not guaranteed or endorsed by the publisher.

## References

[ref1] Alden L. E. Wiggins J. S. Pincus A. L. (1990). Construction of circumplex scales for the inventory of interpersonal problems. J. Pers. Assess. 55, 521–536. 10.1207/s15327752jpa5503&4_10, PMID: 2280321

[ref2] Arcelus J. Haslam M. Farrow C. Meyer C. (2013). The role of interpersonal functioning in the maintenance of eating psychopathology: a systematic review and testable model. Clin. Psychol. Rev. 33, 156–167. 10.1016/j.cpr.2012.10.009, PMID: 23195616

[ref3] Bartholomew K. Horowitz L. M. (1991). Attachment styles among young adults: a test of a four-category model. J. Pers. Soc. Psychol. 61, 226–244. 10.1037/0022-3514.61.2.226, PMID: 1920064

[ref4] Bird T. Tarsia M. Schwannauer M. (2018). Interpersonal styles in major and chronic depression: a systematic review and meta-analysis. J. Affect. Disord. 239, 93–101. 10.1016/j.jad.2018.05.057, PMID: 29990668

[ref5] Bowlby J. (1973). Attachment and Loss: Volume II: Separation, Anxiety and Anger. London: The Hogarth Press and the Institute of Psycho-Analysis, 1–429.

[ref6] Cain N. M. Pincus A. L. Grosse Holtforth M. (2010). Interpersonal subtypes in social phobia: diagnostic and treatment implications. J. Pers. Assess. 92, 514–527. 10.1080/00223891.2010.513704, PMID: 20954053

[ref7] Carver C. S. (1997). Adult attachment and personality: converging evidence and a new measure. Personal. Soc. Psychol. Bull. 23, 865–883. 10.1177/0146167297238007

[ref8] Costa P. T. McCrae R. R. (1985). The NEO Personality Inventory. Odessa, FL: Psychological Assessment Resources.

[ref9] Costa P. T. McCrae R. R. (1992). Neo Pi-R. Odessa, FL: Psychological Assessment Resources.

[ref10] Derogatis L. R. Lipman R. S. Rickels K. Uhlenhuth E. H. Covi L. (1974). The Hopkins Symptom Checklist (HSCL): a self-report symptom inventory. Behav. Sci. 19, 1–15. 10.1002/bs.3830190102, PMID: 4808738

[ref11] Dinger U. Zilcha-Mano S. McCarthy K. S. Barrett M. S. Barber J. P. (2013). Interpersonal problems as predictors of alliance, symptomatic improvement and premature termination in treatment of depression. J. Affect. Disord. 151, 800–803. 10.1016/j.jad.2013.07.003, PMID: 23932793

[ref12] Du T. V. Yardley A. E. Thomas K. M. (2020). Mapping big five personality traits within and across domains of interpersonal functioning. Assessment 28, 1358–1375. 10.1177/1073191120913952, PMID: 32248694

[ref13] Elmi L. M. Clapp J. D. (2021). Interpersonal functioning and trauma: the role of empathy in moderating the association of PTSD and interpersonal functioning. Behav. Ther. 10.1016/j.beth.2021.02.004 (in press). 34452677

[ref14] Eng W. Heimberg R. G. (2006). Interpersonal correlates of generalized anxiety disorder: self versus other perception. J. Anxiety Disord. 20, 380–387. 10.1016/j.janxdis.2005.02.005, PMID: 16564440

[ref15] Girard J. M. Wright A. G. Beeney J. E. Lazarus S. A. Scott L. N. Stepp S. D.. (2017). Interpersonal problems across levels of the psychopathology hierarchy. Compr. Psychiatry 79, 53–69. 10.1016/j.comppsych.2017.06.014, PMID: 28735709 PMC5643217

[ref16] Grant D. M. Wingate L. R. Rasmussen K. A. Davidson C. L. Slish M. L. Rhoades-Kerswill S.. (2013). An examination of the reciprocal relationship between avoidance coping and symptoms of anxiety and depression. J. Soc. Clin. Psychol. 32, 878–896. 10.1521/jscp.2013.32.8.878

[ref17] Gude T. Moum T. Kaldestad E. Friis S. (2000). Inventory of interpersonal problems: a three-dimensional balanced and scalable 48-item version. J. Pers. Assess. 74, 296–310. 10.1207/S15327752JPA7402_9, PMID: 10879357

[ref18] Hartmann A. Zeeck A. Barrett M. S. (2010). Interpersonal problems in eating disorders. Int. J. Eat. Disord. 43, 619–627. 10.1002/eat.20747 19718674

[ref19] Hayden M. C. Müllauer P. K. Andreas S. (2017). A systematic review on the association between adult attachment and interpersonal problems. J. Psychol. Psychother. 7, 1–9. 10.4172/2161-0487.1000296

[ref20] Hepp J. Lane S. P. Carpenter R. W. Niedtfeld I. Brown W. C. Trull T. J. (2017). Interpersonal problems and negative affect in borderline personality and depressive disorders in daily life. Clin. Psychol. Sci. 5, 470–484. 10.1177/2167702616677312, PMID: 28529826 PMC5436804

[ref21] Hilbert A. Saelens B. E. Stein R. I. Mockus D. S. Welch R. R. Matt G. E.. (2007). Pretreatment and process predictors of outcome in interpersonal and cognitive behavioral psychotherapy for binge eating disorder. J. Consult. Clin. Psychol. 75, 645–651. 10.1037/0022-006X.75.4.645, PMID: 17663618

[ref22] Hjemdal O. Hagen R. Solem S. Nordahl H. Kennair L. E. O. Ryum T.. (2017). Metacognitive therapy in major depression: an open trial of comorbid cases. Cogn. Behav. Pract. 24, 312–318. 10.1016/j.cbpra.2016.06.006

[ref23] Hopwood C. J. Wright A. G. Ansell E. B. Pincus A. L. (2013). The interpersonal core of personality pathology. J. Pers. Disord. 27, 270–295. 10.1521/pedi.2013.27.3.270, PMID: 23735037 PMC3675800

[ref24] Horowitz L. M. Rosenberg S. E. Baer B. A. Ureño G. Villaseñor V. S. (1988). Inventory of interpersonal problems: psychometric properties and clinical applications. J. Consult. Clin. Psychol. 56, 885–892. 10.1037/0022-006X.56.6.885, PMID: 3204198

[ref50] Marino C. Vieno A. Moss A. C. Caselli G. Nikčević A. V. Spada M. M. (2016). Personality, motives and metacognitions as predictors of problematic Facebook use in university students. Pers. Individ. Differ. 101, 70–77. 10.1016/j.paid.2016.05.053

[ref25] Martinsen Ø. L. N. H. Nordvik H. Østbø L. (2005). Norske versjoner av NEO PI-R og NEO FFI [Norwegian versions of NEO PI-R and NEO FFI]. Tidsskrift for Norsk Psykologforening 42, 421–423.

[ref26] McEvoy P. M. Burgess M. M. Nathan P. (2014). The relationship between interpersonal problems, therapeutic alliance, and outcomes following group and individual cognitive behaviour therapy. J. Affect. Disord. 157, 25–32. 10.1016/j.jad.2013.12.038, PMID: 24581824

[ref27] Myers S. G. Wells A. (2015). Early trauma, negative affect, and anxious attachment: the role of metacognition. Anxiety Stress Coping 28, 634–649. 10.1080/10615806.2015.1009832, PMID: 25626392

[ref28] Newman M. G. Jacobson N. C. Erickson T. M. Fisher A. J. (2017). Interpersonal problems predict differential response to cognitive versus behavioral treatment in a randomized controlled trial. Behav. Ther. 48, 56–68. 10.1016/j.beth.2016.05.005, PMID: 28077221 PMC5240795

[ref29] Nordahl H. M. Borkovec T. D. Hagen R. Kennair L. E. Hjemdal O. Solem S.. (2018). Metacognitive therapy versus cognitive–behavioural therapy in adults with generalised anxiety disorder. BJPsych Open 4, 393–400. 10.1192/bjo.2018.54, PMID: 30294448 PMC6171331

[ref30] Nordahl H. Hjemdal O. Hagen R. Nordahl H. M. Wells A. (2019). What lies beneath trait-anxiety? Testing the self-regulatory executive function model of vulnerability. Front. Psychol. 10:122. 10.3389/fpsyg.2019.00122, PMID: 30804834 PMC6371045

[ref31] Nordahl H. Nordahl H. M. Hjemdal O. Wells A. (2017). Cognitive and metacognitive predictors of symptom improvement following treatment for social anxiety disorder: a secondary analysis from a randomized controlled trial. Clin. Psychol. Psychother. 24, 1221–1227. 10.1002/cpp.2083, PMID: 28295802

[ref32] Nordahl H. M. Vogel P. A. Morken G. Stiles T. C. Sandvik P. Wells A. (2016). Paroxetine, cognitive therapy or their combination in the treatment of social anxiety disorder with and without avoidant personality disorder: a randomized clinical trial. Psychother. Psychosom. 85, 346–356. 10.1159/000447013, PMID: 27744447

[ref33] Nordahl H. M. Wells A. (2019). Metacognitive therapy of early traumatized patients with borderline personality disorder: a phase-II baseline controlled trial. Front. Psychol. 10:1694. 10.3389/fpsyg.2019.01694, PMID: 31417453 PMC6682682

[ref34] Normann N. Morina N. (2018). The efficacy of metacognitive therapy: a systematic review and meta-analysis. Front. Psychol. 9:2211. 10.3389/fpsyg.2018.02211, PMID: 30487770 PMC6246690

[ref35] Nysæter T. E. Langvik E. Berthelsen M. Nordvik H. (2009). Interpersonal problems and personality traits: the relation between IIP-64C and NEO-FFI. Nord. Psychol. 61, 82–93. 10.1027/1901-2276.61.3.82

[ref36] Papageorgiou C. Wells A. (2001). Metacognitive beliefs about rumination in recurrent major depression. Cogn. Behav. Pract. 8, 160–164. 10.1016/S1077-7229(01)80021-3

[ref37] Quilty L. C. Mainland B. J. McBride C. Bagby R. M. (2013). Interpersonal problems and impacts: further evidence for the role of interpersonal functioning in treatment outcome in major depressive disorder. J. Affect. Disord. 150, 393–400. 10.1016/j.jad.2013.04.030, PMID: 23726776

[ref38] Renner F. Jarrett R. B. Vittengl J. R. Barrett M. S. Clark L. A. Thase M. E. (2012). Interpersonal problems as predictors of therapeutic alliance and symptom improvement in cognitive therapy for depression. J. Affect. Disord. 138, 458–467. 10.1016/j.jad.2011.12.044, PMID: 22306232 PMC3306447

[ref39] Shin K. E. Newman M. G. (2019). Self-and other-perceptions of interpersonal problems: effects of generalized anxiety, social anxiety, and depression. J. Anxiety Disord. 65, 1–10. 10.1016/j.janxdis.2019.04.005, PMID: 31054457 PMC6658327

[ref40] Solem S. Haaland A. T. Hagen K. Launes G. Hansen B. Vogel P. A.. (2015). Interpersonal style in obsessive compulsive disorder. Cogn. Behav. Ther. 8, 1–17. 10.1017/S1754470X15000719

[ref41] Strand B. H. Dalgard O. S. Tambs K. Rognerud M. (2003). Measuring the mental health status of the Norwegian population: a comparison of the instruments SCL-25, SCL-10, SCL-5 and MHI-5 (SF-36). Nord. J. Psychiatry 57, 113–118. 10.1080/08039480310000932, PMID: 12745773

[ref42] Strand E. R. Hagen R. Hjemdal O. Kennair L. E. Solem S. (2018). Metacognitive therapy for depression reduces interpersonal problems: results from a randomized controlled trial. Front. Psychol. 9:1415. 10.3389/fpsyg.2018.01415, PMID: 30131749 PMC6090231

[ref43] Sun X. Zhu C. So S. H. W. (2017). Dysfunctional metacognition across psychopathologies: a meta-analytic review. Eur. Psychiatry 45, 139–153. 10.1016/j.eurpsy.2017.05.029, PMID: 28763680

[ref44] Tonge N. A. Lim M. H. Piccirillo M. L. Fernandez K. C. Langer J. K. Rodebaugh T. L. (2020). Interpersonal problems in social anxiety disorder across different relational contexts. J. Anxiety Disord. 75:102275. 10.1016/j.janxdis.2020.102275, PMID: 32891027 PMC7755155

[ref45] Wells A. (2009). Metacognitive Therapy for Anxiety and Depression. New York: Guilford Press.

[ref46] Wells A. (2019). Breaking the cybernetic code: understanding and treating the human metacognitive control system to enhance mental health. Front. Psychol. 10:2621. 10.3389/fpsyg.2019.02621, PMID: 31920769 PMC6920120

[ref47] Wells A. Cartwright-Hatton S. (2004). A short form of the metacognitions questionnaire: properties of the MCQ-30. Behav. Res. Ther. 42, 385–396. 10.1016/S0005-7967(03)00147-5, PMID: 14998733

[ref48] Wells A. Matthews G. (1994). Attention and Emotion: A Clinical Perspective. Hove UK: Erlbaum.

[ref49] Wilson S. Stroud C. B. Durbin C. E. (2017). Interpersonal dysfunction in personality disorders: a meta-analytic review. Psychol. Bull. 143, 677–734. 10.1037/bul0000101, PMID: 28447827 PMC5507693

